# The Evolutionary History of Renal Surgery and of Temporal Bone Surgery

**Published:** 1924-01

**Authors:** J. Lacy Firth

**Affiliations:** Surgeon to the Bristol General Hospital


*"Scire est nescire, nisi id me
Scire alius sciret."*

JANUARY, 1924.

### THE EVOLUTIONARY HISTORY OF RENAL SURGERY AND OF TEMPORAL BONE SURGERY.

The Presidential Address, delivered on October 10th, 1923, at the opening of the Fifty-first Session of the Bristol Medico-Chirurgical Society.

BY

J. LACY FIRTH, M.D., M.S. Lond., F.R.C.S.,

*Surgeon to the Bristol General Hospital.*

---

### PART I.

In the winter of 1886–87, thirty-seven years ago, when I became a surgical dresser to Mr. Marcus Beck in University College Hospital, and for the first time came into close contact with surgical cases, I was told by one of my chiefs to pay particular attention to a case of nephrectomy. The operation of nephrectomy, it was said, had not been performed many times, and it was important that every case should be carefully observed and recorded.

I have no recollection of the history of the evolution of this operation being given to the dressers. At that stage of professional education one is apt to regard everything one's chief does as being not only the right thing, but the thing universally sanctioned and practised, and not in any way questionable. To the history of how and when operations came to be practised; how, when, and by whom their safety and usefulness were discovered, the average dresser in my time gave no thought. For anything he knew or cared the operations done might have been in vogue since remote antiquity or but recently introduced. What concerned him was to know when and how operations should be done. If he knew that, he could satisfy examiners and would be fit to advise prospective patients.

Looking back to that period, to the absence of historical teaching and to the attitude of students as just portrayed, leads me to ask whether such neglect of history, where such neglect exists, is wise, and whether it should not, as far as possible, be remedied? Would not the story of the evolution of our science and art, judiciously related, a story as it is of the triumph of mind over matter, increase the students' interest in their work, tend to foster in them the spirit of investigation and research, help them to realise the great debt which we of the present owe to the workers of the past—a debt which we can most suitably strive to repay by endeavouring to do something ourselves, that others may make history of that, and learn from us?

I have mentioned that in 1886 I was told that nephrectomy was a comparatively uncommon operation. I have been curious, therefore, to find out how uncommon or common it was at that time, and the sequel will show this. To-day, of course, nephrectomy is regarded as quite an ordinary proceeding. I have often thought that the surgeon who first removed a kidney from a human being must have been a man of venturesome spirit, but I surmised that he might have been led to do it by fairly easy steps. He might have met with a case in which a badly-injured kidney had to be removed to give the patient any chance whatever of recovery, and then successfully removed it, and one naturally thought the operation would be tried on animals first. As a matter of fact it was tried on animals as early as 1670. But even so great an authority as Rayer wrote early in the nineteenth century that it was "madness to dream of extirpating the kidney in a human subject."

The history of the discovery that nephrectomy, nephrotomy, and other operations on the kidney were right and proper undertakings has been outlined by Sir Henry Morris in the Hunterian Lectures given in 1898. He there states :—"The dawn of renal surgery admittedly dates from the 2nd of August, 1869, when Gustav Simon of Heidelberg performed the first nephrectomy." The best account I have found of Simon's operation, and of the considerations which led him to perform it, is contained in a paper by the late A. E. Barker, published in the *London Medico-Chirurgical Transactions*, and read in March, 1880. The case was one where a ureteral fistula in the abdominal wall had resulted from an ovariotomy performed a year and a half previously. The patient's condition was wretched. All treatment had failed. The study of pathology had shown many instances in which one kidney had gradually become useless by slow disease without notable effect on the economy. But Simon was not satisfied that the *sudden* removal of a functioning kidney would be tolerated. There were two risks, the primary surgical one and that from the possible dangerous effects on urinary secretion. He therefore tried experiments on animals and compared the effects of fifteen hysterectomies performed on bitches and fifteen nephrectomies performed on dogs. He found that both operations were equally well borne. Hemorrhage was not found dangerous. The greatest danger was from peritonitis. He lost one dog in five. Also he found that the physiological effects of the operation were not to be dreaded. Months after operation the dogs were in a thriving condition, and when killed the remaining kidney was found hypertrophied.

A dog's kidney weighs more in proportion to the whole body weight than is the case in man, and the dog has to excrete proportionally four times as much urea as a human being. Therefore, argued Simon, the removal of a human kidney was likely to be safer from the physiological point of view than the removal of a dog's kidney. No albuminuria had followed the nephrectomies and no hypertrophy of the heart.

Led by these considerations, Simon removed the kidney of the woman with ureteral fistula, and the operation was successful. It seems clear that Simon was not a reckless adventurer when he performed this first nephrectomy, but, on the contrary, set an example of conscientious carefulness before submitting his patient to the risks of a severe operation which had never before been done on man. His success proved once for all that a single kidney can suddenly take up and perform the work usually done by two kidneys. How long it would have been, but for this success, before nephrectomy became established as a safe procedure we cannot tell, but I believe the delay would not have been a long one, for the conception of the operation was too soundly based on reason to be extinguished by one or two failures when first placed on trial. In surgery one successful innovation counts more than many failures, for it shows that the causes of failure, somehow or other, are avoidable. There can be no advance made in operative surgery without experiment, using that word in its good sense, in the sense of making a trial. Unfortunately, in these trials a fellow being whom we wish to benefit has to take the greatest risk, not the operator, and to pay the heaviest price for failure. The admonition of Sir W. Mitchell Banks, given in his address to the Surgical Section of the British Medical Association at the Annual Meeting held in Bristol in 1894, comes to my mind in this connection, and it will, I think, commend itself to us all. He said :—" Of this I am sure, that when the week's work of the Association shall be finished it will be found that not a single operation, not a single plan of treatment, will have been suggested which any one of us, should the sad necessity arise, would refuse to extend to his own mother, his own wife or his own child." In parenthesis may I whisper that suggestions of treatment, even with the limitations mentioned, would yet vary much in rashness, depending on the temperament of the surgeon, and that there is a possibility of family affection and tenderness sometimes warping sound judgment on such occasions.

In Barker's paper, two interesting cases of early nephrectomy are quoted which shed light on the question, at that time considered important, of the safety of tying the large sympathetic nerves and ganglia which, with the blood vessels and ureter, are present in the renal pedicle, and are in close relationship with the solar plexus. Great shock might reasonably be expected from so doing. The cases show that it does not occur.

One of these cases was that of Professor Brandt :—

An Austrian peasant received a slash with a knife in the left flank on January 3rd, 1873. He lost two or three ounces of blood at the time. A couple of hours later a fit of coughing forced his kidney out of the wound. A friend replaced it, but prolapse occurred again on coughing. Twenty-four hours later the patient was examined by Brandt, who found the prolapsed kidney was slit nearly in half longitudinally and was pouring out clear urine. It was not painful on handling, but when drawn upon pain was felt in the pedicle. Two days later the patient walked to a studio, remained there two hours while being photographed in various positions, and walked back again. On the fourth day Brandt removed the kidney apparently without an anæsthetic. The pedicle was transfixed and tied in two portions and the organ cut away with a knife. Considerable pain was felt during transfixion, much less during removal. No bad symptoms followed. He left the hospital sixteen days later quite well.

The other case worth relating is rather similar. It is recorded by M. Marvaud, a Surgeon-Major in Algeria :—

A young Arab woman was stabbed in the right flank with a yataghan, which dragged the kidney out of the wound. It bled for a short time and then became strangulated. The pedicle was ligatured and the organ allowed to slough off, which it did at the end of some weeks. The patient continued in good health the whole time, the secretion of urine being normal. She was discharged perfectly well two months after admission to hospital.

The next recorded case of nephrectomy, after that of Simon, was that of Gilmore of America. He removed a shrunken fibrous kidney from a woman who was five months pregnant. She recovered without a miscarriage.

In England the first nephrectomy was performed by Arthur E. Durham in 1872, the next by Jessop of Leeds in 1877, and the third by A. E. Barker. Apparently the two papers by Barker, published in 1880 and 1881 respectively, from one of which I have quoted at some length, brought renal surgery prominently before British surgeons, and after they had been published the operation began to be practised frequently.

As an example of the maintenance of good health after nephrectomy I may here cite the case of a man from whom I removed a tuberculous kidney some years before 1914. In that year he was enlisted in the army as an A1 man, and passed through the Gallipoli campaign almost unscathed, only sustaining a slight leg injury. Later he wrote to me from Egypt to tell me how well he had been.

He remarked at the end of his letter that I should be pleased to hear that he had killed two Turks. Needless to say, I read the latter news with mixed feelings: with sorrow that such barbarity had been necessary, but with gladness that my friend had been on the winning side.

The operation of nephrotomy came into vogue about the same time as nephrectomy, *i.e.* about 1880, when Sir Henry Morris demonstrated for the first time that a renal stone could be safely removed by cutting down upon it through a thick layer of renal parenchyma. Before that nephrotomy had only been done when there was a large pyonephrosis pointing in the loin. In 1881 also Hahn described a method of fixing a movable kidney. This was an important advance, as Sir Henry remarks, "because it at once had the effect of putting a stop to nephrectomy for movable kidney which had been recommended and practised by Martin of Berlin."

When Barker's papers were published nephrectomy had been performed by the lumbar route twenty-seven times with eleven deaths, and by the abdominal route sixteen times with eight deaths. The mortality rate had been very high. The total number of cases tabulated was forty-three in 1881. By 1886, when I became a surgical dresser, no doubt many more cases were on record, so that there was perhaps a little exaggeration in the statement made to me that the operation was a very unusual one, an exaggeration I think justifiable to use as a spur to make me perform my duties as a note-taker with enthusiasm and diligence.

To-day the operative mortality of the operation is perhaps about 10 per cent., taking all classes of disease and injury together. The rate varies much with the nature of the trouble for which it is undertaken. In tuberculous disease, limited to one kidney, or to one kidney and the bladder, the rate of mortality is about 3 per cent.; in malignant disease it has been reduced from 76 per cent. to 11 per cent.; but for sarcoma in children the rate is still 25 to 30 per cent.

These improved modern results are largely due to improved methods of diagnosis and a wiser selection of cases, rendered possible by the introduction of cystoscopy, X-ray examinations and perfected methods of estimating the functional capacity or sufficiency of the kidneys. Let us glance at the evolutionary history of those procedures.

### CYSTOSCOPY.

There are two main epochs in the history of the endeavour to illuminate the bladder, the period before and the period after the introduction of electric lighting.

In the first period attempts were made to reflect light by mirrors from an outside source through tubes into the bladder and urethra. Bozzini in 1805 seems to have been the first to make such an attempt with his so-called "Lichtleiter." He was the introducer of endoscopy into medical practice. In 1855 Desormeaux devised an instrument of a much improved type, by which a beam of light was reflected from its source through a convex lens on to a flat mirror with a hole in it. Through this hole the observer looked. The arrangement is like that of the modern bronchoscopes and œsophagoscopes of Killian and Brünings. There is justification therefore for regarding Desormeaux as "the father of endoscopy," as one writer has named him. Many endeavours were made by his successors to improve the apparatus, and Cruise of Dublin devised an endoscope, an improvement on that of Desormeaux, through which something could be seen fairly satisfactorily. But all these early endeavours to construct an apparatus which would render visible the interior of the male bladder were inefficient.

The second epoch in the history begins with the introduction of the electric light and its application to medical apparatus. A dentist of Breslau, Julius Bruck, jun.,[1] was the first to use such a light for medical purposes. He published a book on the subject in 1867. The light employed was an incandescent platinum wire at a white heat. It was used either for direct illumination or for transillumination, so-called diaphanoscopy. He invented a stomatoscope to light up the mouth directly, and an instrument for lighting up the bladder by transillumination. The latter apparatus was introduced into the rectum and a sort of catheter with a glass partition in it was passed into the bladder for the observer to look through. The platinum spiral in both these instruments was surrounded by a jacket in which cold water circulated to keep down the heat. Though the apparatus proved practically useless, Bruck is to be credited with having pointed out the only way by which good illumination of closed cavities in the body can be obtained, that is by introducing the light itself into the cavity.

Twelve years after Bruck, Dr. Max Nitze of Berlin began to produce cystoscopes. Mr. Hurry Fenwick has written as follows[2] :—" To Dr. Max Nitze belongs by right the honour and the credit of introducing the method of employing the electric light in the illumination of the deeper cavities of the body, such as the stomach and bladder, for diagnostic purposes." Mr. Fenwick mentions the rather interesting fact that Nitze was at one time assistant to a Dr. Schramm, of Dresden, who had devised an instrument, using the incandescent platinum wire light as Bruck did, for diaphanoscopy of the ovaries. The light was to be placed in the vagina, so that in a dark room the outline of the ovaries could be seen by the light transmitted through

[1] *Handbuch der Cystoscopie*, Dr. Leopold Casper, 1898, p. 5.

[2] *Electric Illumination of the Bladder and Urethra.*

the abdomen. In thin persons this appears to have been sometimes successful. In Nitze's first cystoscopes the light was of the same kind as that used by Bruck, and of course it was necessary to use a water-cooling apparatus with it. Provision had to be made by means of small tubes running inside the cystoscope to carry the cold water to and from the jacket surrounding the light. These tubes had to be of very small calibre, and therefore the water had to be forced along them under considerable pressure to insure that the circulation would be brisk enough to keep the lamp cool. The whole water circulating apparatus was complicated and cumbrous in a high degree, though arranged with great ingenuity.

In the early seventies the problem of enlarging the field of vision of the cystoscope was solved. Until that problem had been solved the portion of the bladder which could be seen at one look was not much larger than the area of the cross-section of the urethral tube of the instrument. An optician named Beneche, at the instigation of Nitze, considered and solved this problem. With great ingenuity he fixed a combination of lenses into a tube, as lenses are fixed in a telescope, which widened the field of vision. In 1876 Nitze began to work with the Vienna surgical instrument maker Leiter, and together in 1879 they brought out the first "Nitze-Leiter cystoscopes." Two patterns were produced, one for seeing the anterior parts of the bladder with the light shining from the front of the beak, the other with the light shining from the posterior surface. In the former, as in all or most modern cystoscopes, there was fixed a prism to refract the image of the bladder along the tube of the cystoscope and its telescope. The image is seen, by this device, much in the same way as an image is seen in using a laryngeal mirror set at an angle of 45 degrees. As one looks through the instrument one appears to be looking straight at those portions of the bladder which lie in front of the prism. The prism is placed in the shaft of the cystoscope close to the angle where the beak begins.

These 1879 instruments, though received with much enthusiasm when first brought out, and though they really did occasionally give a view of a useful kind into the bladder, soon fell out of use. Sir Henry Thompson showed them in London, but does not seem to have continued to use them. They soon fell out of use because they frequently failed in their purpose for various reasons—either the light was too poor, or the platinum fused, or the water cooling apparatus failed to act. Also they were complicated, needed expert management, and finally were very expensive.

The era of modern cystoscopy really begins with the use of electric glow lamps of the Edison and Swann types in cystoscopes. Patents for such lamps were taken out by Edison in 1879 and 1880, but it was not until 1887 that the incandescent lamp cystoscopes of Nitze, of Leiter and of Mr. Hurry Fenwick appeared. Nitze and Leiter were by this time working separately, and each brought out a cystoscope. Mr. Fenwick devised one combining the advantages of both. The particular suitability of the carbon filament lamps for cystoscopes was due to the fact that the conduction of heat from the filaments in these lamps was very much diminished because they were in a vacuum, the heat conductor, air, having been removed. To-day metal filament lamps are mostly used, which are cooler still, and they can be kept burning in air without getting hot or suffering harm. With their introduction the need for the water-cooling apparatus was abolished, a very great improvement.

We have seen then that in 1887 the problem of cystoscopy was finally solved. Soon after that date irrigation cystoscopes were invented and then catheterising cystoscopes.

The latter enable the surgeon to pass catheters of small calibre through the cystoscope in special channels until the tips emerge into view in the bladder and can be pushed on into the ureters. By means of an ingeniously contrived lever the emerging catheter can be tilted backwards and forwards so that its tip passes into the openings of the ureters at the most suitable angle for entry. This lever was devised by Albarran, of Paris. The optical parts of the instrument have been improved by the use of special prisms which produce " by refraction only successive inversions of the image " so that an erect image is presented to the observer's eye. In the original Nitze cystoscope the image seen was an inverted one.

When I left University College Hospital in 1890 the use of cystoscopes there was beginning, but I remember hearing students joking about what so and so said he had seen with them, though subsequent exploration had shown that the things said to have been seen were not there. I served in hospitals at Reading and at Bradford, 1890 to 1893. Neither institution possessed a cystoscope in those days. In June, 1893, I came to the Bristol General Hospital as House Surgeon, and found there two cystoscopes of Leiter's manufacture, but I also found that no member of the staff had ever seen anything through them. Nor could I !

The first time I saw anything through a cystoscope was when I had a few private lessons in cystoscopy from Dr. Kapsammer, of Vienna. This was about the year 1900. Dr. Kapsammer advised me to get a cystoscope from the firm of Löwenstein, of Berlin. I did this, and possess the instrument still, and it is still serviceable, though I now use later models which give an upright image.

I think it was the following year that I visited Berlin and had instruction in cystoscopy from Dr. Knorr, who had a gynæcological clinic. There, in the post-graduate course, one had an opportunity of practising catheterisation of the ureters. Sometime previous to these continental visits I remember Mr. Percy, of Down Brothers, showing me a catheterising cystoscope, and the surprise I felt, almost amounting to incredulity, on hearing that such a feat as catheterising the ureters in the male was possible. To-day I consider the procedure either quite easy or quite impossible. All turns on whether you can see the ureteral openings well or not. That depends on the condition of the bladder with regard to fasciculation, pouching, deformity from pressure, or other cause, and, of course, upon the possibility of getting a sufficient quantity of clear fluid retained in the organ.

It is hardly necessary for me to indicate how cystoscopy and catheterisation of the ureters help in the diagnosis and treatment of renal disease. They are, of course, the chief means by which we determine whether there is only one kidney present or more than one; which kidney is the diseased one or the more diseased of the two; and whether, if nephrectomy is contemplated, we can rely on the functional capacity of the kidney which is to be left behind being sufficiently good to maintain health. It is naturally a golden rule in renal surgery that you must not remove a kidney unless you can be reasonably sure that you will leave behind a second kidney of good or fair efficiency. I have myself seen two deaths result from breaking that rule. Again, in tuberculous disease of the urinary organs it is most important to discover whether the disease is limited to one kidney or not. The cystoscope, when it can be used, saves the patient from having to submit to an exploratory operation on both kidneys to settle this question. But I am preaching to the converted, and need say no more, for all surgeons feel that the cystoscope is well-nigh indispensable.

I consider the modern cystoscope to be an instrument of beauty—it has beauty of appearance, beauty in design, beauty in its adaptability for its purposes and its use yields the observer many pictures of beauty. An illuminated Cheddar Cave with glittering stalactites has beauty, sea-shore pools with the graceful forms of life found therein have beauty, and, to my mind, some of the things seen with the cystoscope are equally beautiful, though their significance, alas! is too often of a sinister kind.

### RADIOGRAPHY.

The next innovation of our time which helped the development of renal surgery was the use of X-rays in the examination of patients, and latterly the combined use of the catheterising cystoscope and X-rays for pyelography and cystography, an opaque meal, as it were, being given to the kidney through the catheter in the ureter, and this followed up by skiagraphy. By the latter means a more or less clear map of the calyces, pelvis and ureter of the kidney is given, showing any changes of anatomical form which may be present.

### RENAL EFFICIENCY TESTS.

Lastly, in our consideration of the means by which the results of nephrectomy have been so greatly improved in modern times we have to notice the introduction of new methods of estimating renal sufficiency.

These tests are of three classes: firstly, tests of excretion applied to urine passed in the normal way; secondly, tests of the urine after artificial stimulation of the kidneys; and thirdly, "retention tests" applied to the blood.

The *first* class includes the quantitative estimation of various substances found in the urine and tests by cryoscopy, and by measurement of the electrical resistance of the urine.

The *second* class includes the estimation of the contents of the urine after ingestion or injection into the tissues of certain substances or drugs such as urea (Albarran, 1905, McCaskey, 1913–14) ; phloridzin, which is excreted as sugar (Von Mehring, 1882) ; phenolsulphonaphthalein (Rowntree and Geraghty, 1910) ; indigo-carmine (Haidenhain, 1874) and Voelcker and Joseph, 1903) ; methylene blue (Casper, 1892).[1] In using these tests either quantitative tests or tests based on the time at which the substances appear in the urine are applied.

The *third* class includes quantitative tests applied to blood to estimate the urea content or acidosis or chloride content.

In surgical practice we require knowledge either of the capacity of both kidneys taken together, for example, before prostatectomy, or we require knowledge of the functional capacity of each kidney separately, for example, before nephrectomy.

Many of us content ourselves before prostatectomy with the urea concentration test applied to the urine, and an estimation of the urea content of the blood, and before nephrectomy by noting the time when indigo-carmine begins to escape from the ureters and the intensity of the colouration, as seen with the cystoscope, after injection of the dye either into a muscle or into a vein.

The following lantern illustrations of the evolution of cystoscopy were shown :—

(1) Desormeaux's Endoscope of 1855. (2) and (3) Cruise's Endoscope of 1865. (4) Bruck's Diaphanoscope of 1867. (5) The Nitze-Leiter Platinum-Loop Cystoscope of 1879. (6) The Nitze Glow Lamp Cystoscope of 1887. (7) The Optical Effect of the Prism. (8) The Telescopic Arrangements. (9) and (10) The Method of Examination of the Anterior and Posterior Walls of the Bladder. (11) and (12) Portraits of Gustav Simon (b. 1824, d. 1876) and of Max Nitze (b. 1848, d. 1906).

[1] "Tests of Renal Efficiency," by Frank Kidd, *Lancet*, 1922, i. 788.

